# Reductive Mobilization of Iron from Intact Ferritin: Mechanisms and Physiological Implication

**DOI:** 10.3390/ph11040120

**Published:** 2018-11-05

**Authors:** Fadi Bou-Abdallah, John J. Paliakkara, Galina Melman, Artem Melman

**Affiliations:** 1 Department of Chemistry, State University of New York at Potsdam, Potsdam, NY 13676, USA; paliakjj199@potsdam.edu; 2 Department of Chemistry & Biomolecular Science, Clarkson University, Potsdam, NY 13699, USA; gmelman@clarkson.edu

**Keywords:** ferritin, iron mobilization, chaotropes, flavin nucleotide, electron transfer, kinetics

## Abstract

Ferritins are highly conserved supramolecular protein nanostructures composed of two different subunit types, H (heavy) and L (light). The two subunits co-assemble into a 24-subunit heteropolymer, with tissue specific distributions, to form shell-like protein structures within which thousands of iron atoms are stored as a soluble inorganic ferric iron core. In-vitro (or in cell free systems), the mechanisms of iron(II) oxidation and formation of the mineral core have been extensively investigated, although it is still unclear how iron is loaded into the protein in-vivo. In contrast, there is a wide spread belief that the major pathway of iron mobilization from ferritin involves a lysosomal proteolytic degradation of ferritin, and the dissolution of the iron mineral core. However, it is still unclear whether other auxiliary iron mobilization mechanisms, involving physiological reducing agents and/or cellular reductases, contribute to the release of iron from ferritin. In vitro iron mobilization from ferritin can be achieved using different reducing agents, capable of easily reducing the ferritin iron core, to produce soluble ferrous ions that are subsequently chelated by strong iron(II)-chelating agents. Here, we review our current understanding of iron mobilization from ferritin by various reducing agents, and report on recent results from our laboratory, in support of a mechanism that involves a one-electron transfer through the protein shell to the iron mineral core. The physiological significance of the iron reductive mobilization from ferritin by the non-enzymatic FMN/NAD(P)H system is also discussed.

## 1. Introduction

Iron is an essential element required for virtually all living organisms and is involved in numerous biological reactions including respiration, oxygen transport, electron transfer, oxidative metabolism, deoxyribonucleotide synthesis, and gene regulation [1,2]. Due to the wide range of redox potentials of Fe^3+^/Fe^2+^ metal complexes (from approximately −500 to +600 mV), iron cations are indispensable cofactors for numerous enzymes catalyzing oxidation–reduction reactions [3,4,5]. Almost all iron cations in living cells are tightly bound to metalloproteins, leaving less than one percent as “labile iron pool” in the cytosol. The pool contains “free” or loosely bound iron cations predominantly in the reduced Fe^2+^ form. The labile iron pool serves as the main source of iron for the synthesis of iron proteins. However, the presence of labile ferrous ions constitutes a considerable problem for all aerobic organisms, due to their involvement in Fenton reaction in the presence of hydrogen peroxide, an inevitable byproduct of cellular respiration. The resultant hydroxyl radicals react rapidly with cellular biopolymers, including proteins, lipids, and nucleic acids, resulting in their oxidation and loss of functionality. In living cells iron(III) cations produced via Fenton reaction can be rapidly reduced back to iron(II) cations, leading to a continuous cycle of hydroxyl radicals (HO▪) production. To avoid this dangerous cycle of hydroxyl radicals formation, the concentration of labile iron is regulated by a strict control of the rates of iron uptake and mobilization in iron transport and storage proteins. While cellular iron trafficking is suggested to occur via iron chaperons [6], the reported affinity of such chaperones to iron cations is relatively low, suggesting an easy iron exchange mechanism with the labile iron pool.

**Figure 1:**
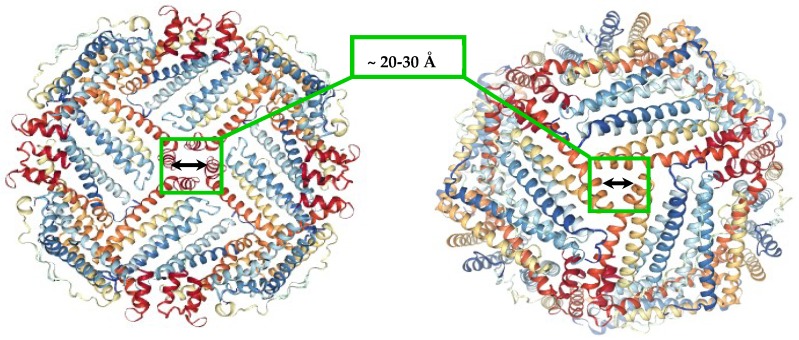
Ferritin molecule (PDB 1R03) with highlighted hydrophobic four-fold (**left**), and hydrophilic three-fold channels (**right**), that allow the transport of small molecules and ions to the inner cavity.

The most efficient way to protect cells from the toxic effect of elevated levels of labile iron cations is ferritin, a ubiquitous and well-characterized iron storage and detoxification protein. A single ferritin molecule is capable of sequestering up to thousands of iron atoms, in the form of insoluble inorganic ferrihydrite core [7,8,9,10]. In bacteria, plants, fish, and amphibians, ferritins are generally homopolymers composed of H-type subunits, while in animals, ferritin is a heteropolymer, typically consisting of 24 subunits of two types, H and L (Figure 1) [10,11]. The resultant protein nanocage, separating the inner cavity of ferritin from the outside environment is remarkably stable in the pH range of 3–9, and temperatures up to 80 °C. Small molecules and various cations have been shown to enter the ferritin interior through eight narrow hydrophilic three-fold channels, and possibly through six hydrophobic four-fold channels as well [10]. The natural Fe^2+^ cations rapidly diffuse through the ferritin shell, where it is oxidized to insoluble iron(III) cations to form the inorganic ferrihydrite core. In mammals, the H-subunit is responsible for the rapid oxidation of Fe(II) to Fe(III) by molecular oxygen (or by hydrogen peroxide) at a dinuclear center, while the L-subunit helps iron clearance from the center in support of iron nucleation and mineralization [12,13,14,15]. Ferritin composed exclusively of L-subunits is still capable of oxidizing iron(II) cations, although at a much lower rate. 

Bacteria can express at least four types of ferritin-like proteins with iron storage capability, two of which (FtnA and FtnB) are structurally similar to conventional ferritins, in term of subunit arrangement and the formation of nanocages (i.e., composed of 24 identical H-like subunits) [16]. The third type, bacterioferritin (Bfr), contains up to 12 protoporphyrin IX heme groups sandwiched between two-fold symmetry-related subunits, where they are bound covalently to methionine groups. The fourth ferritin type is called Dps (or DNA-binding protein from starved cells), contains 12 identical subunits, and thus has half the size of typical ferritins [17,18]. All ferritin types (except FtnB) possess rapid ferroxidase activity, and form ferrihydrite iron cores similar to conventional ferritins [19,20]. In bacteria, iron release from ferritin occurs through reduction of the inorganic iron core by bacterioferritin associated ferredoxin (Bfd) [19,20]. Transfer of electron from Bfd to the iron hydroxide core is mediated by bacterioferritin-bound heme molecules, most likely through electron transfer mechanisms [21]. However, very little is known about how the mechanisms of oxidative storage and reductive mobilization of iron are regulated in bacteria.

## 2. Brief Overview of Iron Reductive Mobilization from Intact Ferritin

While iron uptake and oxidation in conventional ferritin is relatively well understood, the mechanism of iron mobilization is rather controversial [22,23,24,25,26]. It is well established that the major pathway of iron mobilization from ferritin is based on proteolytic degradation, which involves transport of ferritin molecules into the lysosomes, followed by dissolution of the exposed iron hydroxide core [22,27,28]. The half-life of ferritin circulation in eukaryotic cells is about 19–24 h, under conditions of iron deficiency, but is much longer in iron-abundant environments [29], suggesting a tightly regulated cellular iron mobilization, although the exact mechanism remains unclear. We still do not know if proteolytic degradation of ferritin is the only mechanism by which iron is released from the protein, and whether other auxiliary iron mobilization mechanisms for the immediate access of cells to iron exist. Multiple reports on the reductive mobilization of iron cations from intact ferritin appeared in the literature, demonstrating the easy reduction of the iron core by a variety of one electron reducing agents, including flavin mononucleotide [30,31], ascorbate [32,33], sodium dithionite [34] and superoxide [35,36]. The rate of reduction is strongly dependent on the reduction potential of the electron donor [30]. In contrast, while two electron reductants, such as NADH [23] and glutathione [31], possess strongly negative reduction potentials, they generally reduce ferritin very slowly. One notable exception is thioglycolic acid [37], which reacts much more rapidly with the ferritin iron core, and is commonly used in the preparation of iron-free apoferritin. It has been shown that FtnA ferritin, from Pseudomonas aeruginosa, undergoes reductive mobilization of iron(III) by treatment with NADPH in the presence of ferredoxin-NADP^+^ reductase and 2,2′-bipyridine; however, the physiological relevance of this process is not clear, even for anaerobic conditions [19,38].

**Figure 2:**
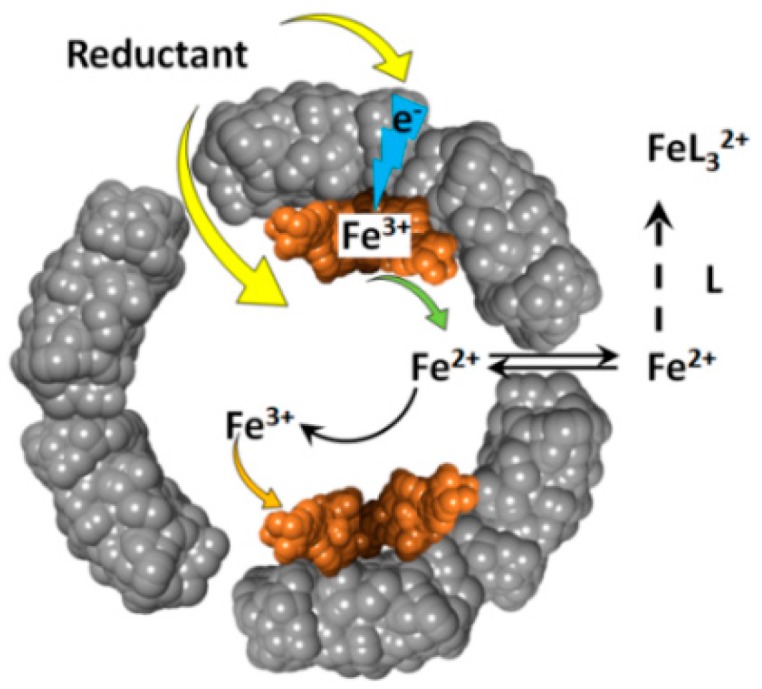
Schematic depiction of the oxidative deposition and reductive mobilization of iron in ferritin.

The reduction of ferritin iron by these reducing agents, produces soluble Fe(II) cations that exit the ferritin internal cavity, through the eight hydrophilic three-fold channels, and join the cellular labile iron pool (Figure 2). The newly formed Fe(II) cations can be easily re-oxidized to Fe(III) , through the catalytic activity of the ferritin’s ferroxidase centers. The rate of ferrous ions re-oxidation is very high [39,40], and their concentration is likely very low, and difficult to observe. This difficulty in detecting reduced iron(II) cations, can be resolved by the addition of iron(II) specific chelate ligands, such as 1,1’-bipyridine, phenanthroline, or ferrozine. The presence of excess chelators prevents the re-oxidation of iron(II) cations, and provides straightforward spectrophotometric detection of the resultant Fe(II)-chelate complexes, which typically have high (~10^3^–10^4^ M^–1^cm^–1^) absorbance metal-to-ligand-charge-transfer (MLCT) bands [41]. 

Amongst the most potent ferritin reducing agents in cell free systems are reduced flavins, including FMNH2 and FADH2 [31]. Reduced flavins can be prepared by chemical reduction of flavins, under oxygen-free conditions [31], or by in-situ reduction of NADH or NADPH. The latter reaction can be catalyzed by NAD(P)H:flavin oxidoreductase [30], or can proceed without enzymatic catalysis at higher concentrations of flavins and NADH [42]. Because a significant concentration (several mM) of flavins is commonly found in the cytosol of living cells, reduced flavins are possible candidates for the hypothetical reductive mobilization of iron cations from intact ferritins [30,31], although the physiological relevance of this mechanism is far from being obvious. Free reduced flavins, such as FMNH2 and FADH2, that are not protein-bound, are rapidly oxidized by molecular oxygen [43]. While FMN can be reduced back to FMNH2 by cellular NAD(P)H:flavin oxidoreductases [30], the rate constants for the re-oxidation of reduced flavins by molecular oxygen are very high [43], suggesting that the stationary concentrations of reduced flavins is extremely low. Unfortunately, there have been multiple studies on the reduction of ferritin iron by NADH without any consideration of the amount of dissolved oxygen present in solutions. 

## 3. Iron Mobilization by the FMN-NADH System

**Figure 3:**
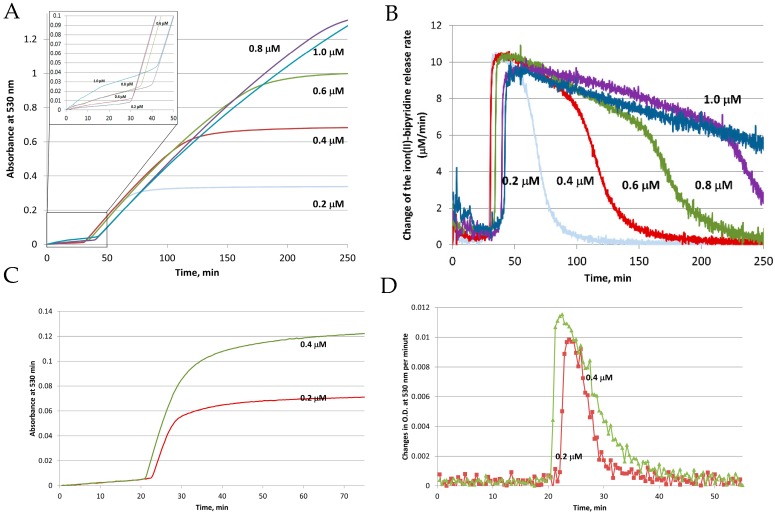
Reductive mobilization of iron from HosF containing 2250 Fe/shell. Conditions: 0.2–1 μM ferritin, 2 mM NADH, 200 μM FMN, 2 mM 2,2′-bipyridine, 2650 U/mL catalase, at pH 7.0 and 22 °C. (**A**) Absorbance change of [Fe(bipy)_3_]^2+^ as a function of time. (**B**) Change of the iron(II)–bipyridine release rate versus time for different concentrations of HosF. (**C**,**D**) Reductive mobilization of iron from human recombinant heteropolymer ferritin (0.2 and 0.4 μM) loaded with 500 Fe/protein, in the presence of 2 mM NADH, 200 μM FMN, 2 mM 2,2′-bipyridine, 2650 U/mL catalase, at pH 7.0 and 28 °C. (**C**) Absorbance change of [Fe(bipy)_3_]^2+^ as a function of time. (**D**) Change of the iron(II)–bipyridine release rate versus time, for different concentrations of heteropolymer ferritin. Reprinted with permission from Ref. [23].

To clarify the influence of each player on the reductive mobilization of iron from intact ferritins, we studied the kinetics of iron reduction by the FMN-NADH system, using horse spleen ferritin (HosF), and recombinant human heteropolymer apoferritin (~21H- and 3L-subunits, referred to as HuHLF), under a tight control of O_2_ concentration [23]. In both cases (Figure 3), the iron release kinetics showed a biphasic behavior. The initial lag phase was followed by a second phase featuring a rapid iron release kinetic. The initial lag phase exhibited a small increase in absorption at 530 nm, indicating a slow iron release process. The second phase is characterized by a dramatic increase in the iron release rates, which depend on the concentrations of FMN and NADH, but not on the concentration of ferritin. The linear increase in the rates of iron mobilization suggests that the generation of FMNH2 by the FMN-NADH system, is the rate-limiting step under these conditions. Notably, the gradual decrease in the rates of iron mobilization from ferritin (as observed towards the end of the reactions in Figure 3 (right), is due to the depletion of the iron(III) hydroxide core, such as the reduction of the ferritin iron core becomes the rate limiting step [23]. 

The significant release of iron from ferritin does not occur until essentially all the dissolved oxygen has been depleted, as observed in Figure 3 (left). The duration of the lag phase depends on the concentration of dissolved oxygen, as well as the concentrations of NADH and FMN, but not on the concentration of ferritin. This indicates that most of the FMNH2, produced from the reaction between FMN and NADH, is re-oxidized back to FMN by dissolved molecular oxygen, without an appreciable release of iron. This re-oxidation reaction is very fast, even at a low concentration of dissolved oxygen, which can be expected, since the rate of FMNH2 oxidation by oxygen is much faster than that of iron reduction [43].

**Figure 4:**
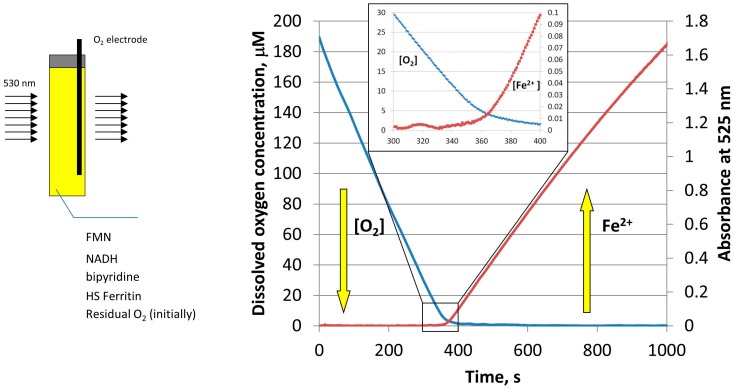
Schematic of the simultaneous monitoring of the dissolved oxygen concentration (blue curve), and light absorption of Fe(II)–bipyridine complex at 530 nm (red curve), during the reductive release of iron from HosF (0.6 μM), in the presence of 2 mM NADH, 2 mM FMN, and 2 mM 2,2′-bipyridine. Reprinted with permission from Ref. [23].

To further determine the effect of oxygen on the amount and rate of iron release from ferritin, the experiments were repeated, while simultaneously monitoring the concentration of molecular oxygen by a MI 730 Clark oxygen microelectrode [23]. As shown in Figure 4, the concentration of dissolved oxygen decreases sharply during the induction period (lag period), without any noticeable iron release. Only when oxygen concentration reaches a level of ~5 µM, that iron reduction and chelation by bipyridine is observed [23]. Because oxygen concentration in this experiment is well below typical normoxic conditions, the results presented in Figure 4 suggest that free reduced flavins are unlikely to serve as ferritin-reducing agents, in normally oxygenated cells. Significantly, under these conditions, the rates of iron mobilization from ferritin would be too low, even in the absence of inevitable re-oxidation of newly formed iron(II) cations. It is also highly improbable that the rates of iron mobilization are strongly dependent on cells oxygenation level, which is known to vary widely under different circumstances.

## 4. Iron Mobilization by Reduced Flavins

**Figure 5:**
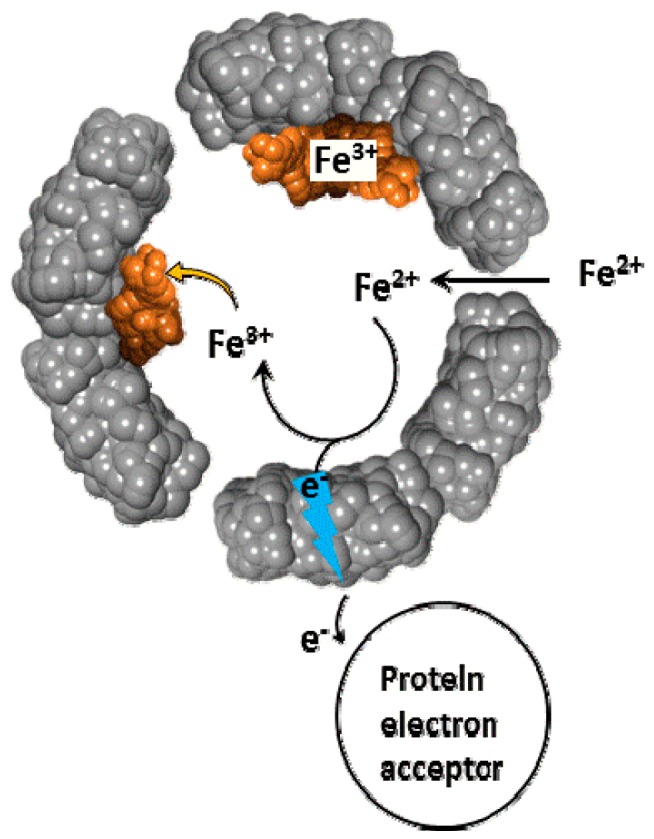
Schematic of Fe^2+^ cations diffusion and electron transfer across the ferritin shell, followed by Fe^2+^ oxidation and deposition.

Reduction of ferritin iron cores by reduced flavins (and other reductants) has been the subject of intense investigation [23,25,26,30,31,44,45,46]. Iron reduction can proceed through two possible mechanisms that involve either a passive diffusion of reducing agents across the ferritin’s shell [30], or the transfer of electrons through specific pathways along the protein shell [46] (Figure 5). Literature evidence in support of the diffusion mechanism is based on two experiments, the first of which employed agarose-bound FMNH2, produced by a reaction of agarose-bound FMN with NADH [30]. The inability of agarose-immobilized FMNH2 to induce reductive mobilization of iron cations from horse spleen ferritin [31], was considered as proof of the inability of FMNH2 to diffuse to the protein’s interior, and that ferritin iron reduction reactions can only proceed through FMNH2 diffusion into ferritin. The problem with this experiment is that it failed to account for possible changes in the reactivity of reduced flavins caused by agarose binding. Alternatively, derivatization of agarose by FMN may occur at the surface of the agarose grains, but also inside the agarose beads, which may not be available for ferritin diffusion and subsequent iron reduction [31]. 

The second piece of evidence is the observation of increased rates of iron reductive mobilization in the presence of chaotropes, or specific peptide molecules in solution [42,47,48]. It was shown that the presence of 1 M urea resulted in a dramatic (more than five-fold) increase in the rates of iron reduction in ferritin, by the FMN-NADH system, in the presence of 2,2′-bipyridine [42]. The data were interpreted as a weakening of the ferritin inter-subunits interactions, which resulted in an enlarged diameter of the three-fold or four-fold axes, thus facilitating the diffusion of FMNH2 molecules into the ferritin interior. Furthermore, certain short peptide sequences (i.e., HSNTYYFPKGG) were shown to exhibit more than three-fold increased iron release rates from ferritin [48]. These later results, however, cannot be easily interpreted, given that the rates of iron reduction in ferritin by the non-enzymatic FMN-NADH system are limited by the rate of FMNH2 production [23], and not by the rate of iron reduction. Additionally, these experiments did not disclose the initial amount of dissolved oxygen in solution, or the possibility of oxygen diffusion inside the reaction cell, which, as reported above, dramatically alters the amount, and rates of iron release from ferritin. 

Evidence for electron transfer reactions across the protein shell are based on the rapid oxidation of iron(II) cations inside the ferritin cavity, under anaerobic conditions, by large electron acceptors, such as plastocyanin, cytochrome c, or stellavyanin [26,46,49]. Because of the large size of these proteins, and their inability to diffuse across the protein shell, iron oxidation reactions can only be explained by electron transfer, from iron(II) cations to these electron acceptors. In further support of this mechanism, iron oxidation and deposition reactions were also observed in L-subunit ferritins, which lack ferroxidase centers, and therefore ferroxidase activity [49]. Such a long range electron transport across the 2 nm protein shell, can be facilitated by the redox activity of the protein shell [46]. 

**Figure 6:**
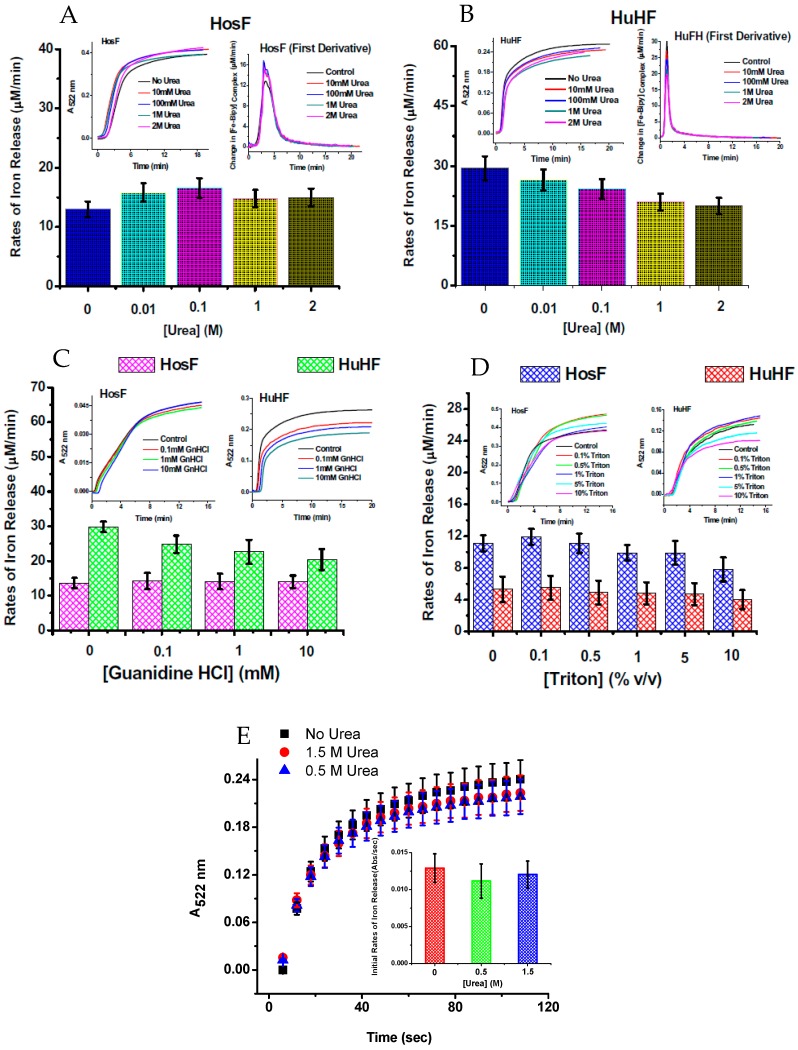
Rates of iron release from HosF and HuHF by the FMN-NADH system (**A**–**D**), and by FMNH_2_ (**E**), in the absence or presence of different concentrations of chaotropes. In both experiments, the presence of urea does not affect the rates of iron mobilization. Reprinted with permission from Ref. [25].

To further understand the iron reductive mobilization from ferritin, and differentiate between the two mechanisms (diffusion vs. electron transfer), more data is needed, using different types of ferritin (i.e., heteropolymer horse spleen ferritin (HosF), homopolymer human H-chain or L-chain ferritin (HuHF or HuLF), and heteropolymer human H/L ferritin (HuHLF)). Recent experiments from our laboratory [25], using the FMN-NADH system (or reduced flavin FMNH2), were performed with HosF and HuHF, under controlled concentration of oxygen. In contrast to literature reports, our data showed no (or insignificant) difference in the reaction rates, in the presence of 0–2 M urea, guanidine chloride, or Triton X100 (Figure 6), suggesting that the kinetics of iron release from ferritin by flavins are independent on the type, or amount of chaotropes present. Additional experiments performed under strict anaerobic conditions, using solutions of FMNH_2_ prepared by catalytic hydrogenation with Adams catalyst, revealed much faster reduction reactions, with no effect from chaotropes on the rates of the iron mobilization reactions [25]. 

The above results suggest that the earlier studies may have been inaccurately interpreted, and that the diffusion mechanism should be revisited. If FMNH2 is able to diffuse into the ferritin interior and reduce the iron(III) hydroxide core, then the produced FMN should be able to diffuse out of ferritin. Notably, the diffusion of various molecules of similar size to FMN, including cisplatin [50], curcumin [51], or anthocyanin [52] is known to be extremely slow, suggesting that diffusion of FMNH2 is unlikely to be responsible for the observed fast rates of iron reduction. To further explore the origin of these discrepancies, we carried out an experiment to unambiguously demonstrate that, neither FMN, nor FMNH2, both of which possess virtually identical steric size, can diffuse through the ferritin shell [25]. All of the aforementioned molecules, including FMN, can be trapped inside the inner cavity of ferritin, by first denaturing the protein down to its individual subunits at pH 2, followed by their re-assembly at neutral pH, and dialysis [50,51,52,53]. The resulting ferritin-FMN entrapped molecules, were found to be stable for days, without any sign of diffusing out. These results clearly indicate that the ferritin channels are too small for FMN (or FMNH2) diffusion, implying that electron transfer is most likely the pathway by which ferritin iron core is reduced by flavins. Given the thickness of the protein shell (~2 nm), it is reasonable to assume that these electron transfer processes are not arbitrary, but rather a conserved evolutionary feature of ferritin, accompanied by specific protein structural arrangements. Interestingly, recent reports demonstrated the importance of electron transfer reactions, carried by three aromatic residues surrounding the diiron center of *E. coli* bacterial ferritin in the formation of the iron mineral core [54,55].

We propose that these electron transfer pathways might be physiologically important processes for iron reductive mobilization from ferritin. However, as discussed above, FMNH2 is an unlikely electron donor candidate in cells. Protein-bound reduced flavins are more stable complexes in the presence of oxygen, and could serve as potential reducing agents, similarly to the ferredoxin-bacterioferritin complex [19,21], but this is purely speculative at the moment and requires further investigation. Some exogenous one-electron reducing agents, with less negative reduction potential than FMNH2, were reported to reduce ferritin iron cores in the presence of oxygen [56]; however, these measurements were conducted without the continuous monitoring of oxygen concentration in solution, and require further clarification.

## 5. Iron Mobilization by Other Reducing Agents

It has been reported that ascorbate and glutathione are capable of mobilizing iron(II) cations from ferritin [31]. While the rates of iron reduction were substantially lower than with reduced flavins, the high intracellular concentration (several mM) of the reduced forms of ascorbate and glutathione, could be sufficient for significant mobilization of iron from cellular ferritin, under physiological conditions. Surprisingly, under anaerobic conditions, iron core reduction by ascorbate and glutathione in horse spleen ferritin comes to a complete stop only after ~17–18% of the total iron has been reduced, in direct contrast with reduced flavins and dithionite, where complete iron mobilization was observed. The sudden halt of iron mobilization cannot be the result of a thermodynamic equilibrium, since the reduction potential of both ascorbate and glutathione is much more negative than that of iron(III). Damaged horse spleen ferritin molecules that cannot protect the inorganic iron core from reducing agents, is one possible explanation for this partial iron mobilization. If this were true, then iron(III) cations in undamaged ferritin can NOT be reduced by ascorbate and glutathione under anaerobic conditions, at least in the absence of other agents. In that regard, it is interesting to compare the iron reducing ability of glutathione, with that of thiolactic acid, which is known to induce complete iron mobilization from ferritin. This clear difference in outcome can be rationalized by the small size of thiolactate molecules, and their ability to easily diffuse through ferritin channels, in contrast to glutathione. Under anaerobic conditions, iron mobilization from ferritin by ascorbate is extremely slow, but the presence of oxygen, iron, or copper cations causes a dramatic increase in the rates of iron reduction [57,58]. This effect can be ascribed to the formation of different redox species, most likely free radical species [59].

Superoxide anion radicals were also reported to induce reductive mobilization of iron from ferritin [35,36]. Because superoxide anions are continuously produced by mitochondria, as a byproduct of oxidative phosphorylation, its tiny size would allow its fast diffusion into the ferritin interior, and the subsequent reduction of the inorganic iron core. However, the physiological importance of this process is questionable, since the stationary concentration of superoxide anions is very low (10^–10^ M) , due to the presence of cellular superoxide dismutases [60]. 

Other compounds reported to induce reductive mobilization of iron from ferritin, include polyphenols [61,62], which are abundant in certain foods. While too large to diffuse inside ferritin, they have the ability to donate one electron, and thus reduce the iron core through electron transfer, but more studies are needed to confirm these electron transfer reactions. The challenges of these studies is that these reactions are often accompanied by the auto-oxidation of polyphenol compounds at neutral pH, giving rise to colored products that absorb light at the same wavelength as iron(II)-(bipy)_3_ complexes, and other commonly used Fe(II)-complexes [63].

## 6. Iron Mobilization by Specific Iron(III) Chelating Agents

**Figure 7:**
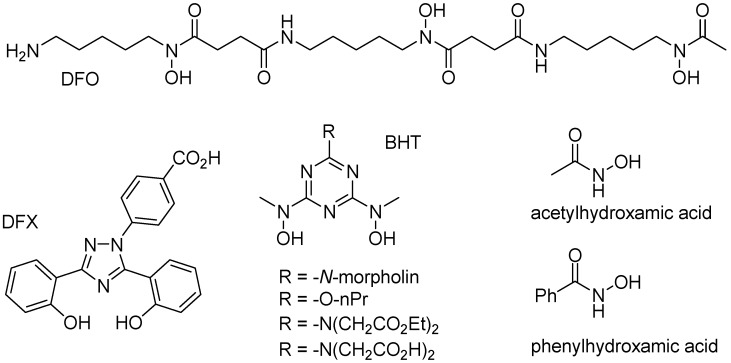
Structures of specific iron(III) chelators.

Iron is a typical “hard” metal capable of binding to a variety of “hard” chelate ligands, including catechols, carboxylates, phosphates, and hydroxamates. Several endogenous chelate ligands of that type, that exist in millimolar concentration in cells, include citrate and ATP [64]. Several other chelate ligands (i.e., DFO, DFX, and BHT; Figure 7), with very high affinity to iron(III) cations have been developed for the treatment of iron overload diseases, such as beta-thalassemia [1,65]. While citrate or EDTA are unable to significantly mobilize iron from ferritin, simple hydroxamates, such as acetyl- or benzoylhydroxamic acid (Figure 7), are able of slowly mobilizing iron from ferritin, presumably because their small size allow them to diffuse across the ferritin shell, and remove iron cations [66]. 

**Figure 8:**
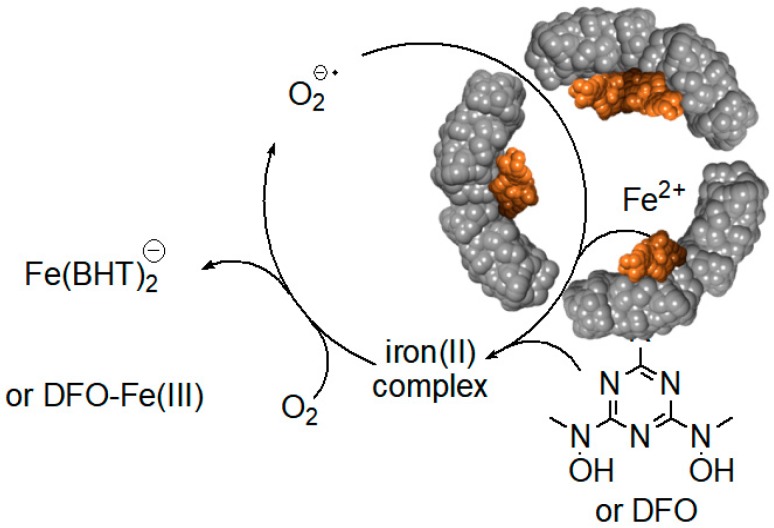
Superoxide mediated mobilization of iron(III) cations from the ferritin iron core by BHT (or DFO) chelators.

Surprisingly, relatively high rates of iron(III) mobilization were observed with large molecules, such as DFO and BHT [24]. These molecules, and their respective 1:1 or 2:1 iron(III) complexes, have molecular sizes too large to diffuse through ferritin channels. The mechanism and the iron release kinetics by these molecules are rather complicated, and strongly depend on the concentration of dissolved oxygen, and the presence of antioxidants, such as mannitol, urea, and superoxide dismutase [24]. Nonetheless, we have proposed a plausible mechanism that is mediated by superoxide anion radicals, which can readily diffuse into the ferritin interior to reduce the iron core, allowing the reduced and soluble iron(II) cations to exit out of ferritin, and be chelated by DFO or BHT ligands [24]. Because of the highly negative reduction potential of the Fe(II)-DFO, and the Fe(II)-BHT complexes (below −0.4 V vs. hydrogen electrode), they act as powerful electron donors capable of reducing dissolved molecular oxygen, thus replenishing superoxide anions (Figure 8). This theoretical cyclic process requires only a catalytic amount of superoxide anions, since they are regenerated after oxidation of the released iron(II) cations to iron(III). However, superoxide anions are unstable species, and can undergo disproportionation. One possible source for the regeneration of superoxide anions is iron(II) hydroxides, embedded within the iron(III) hydroxide core forming a magnetite phase [67]. Reduction of magnetite by one superoxide anion can produce two iron(II) cations, instead of one iron(II) cation in the case of a ferrihydrite phase (composed of only iron(III) hydroxides), thus continuously “breeding” superoxide anions.

This unusual iron reduction mechanism is highly specific for exogenous chelators, possessing very high affinity to iron(III) cations, and is unlikely to be physiologically relevant for cells not exposed to these ligands. Endogenous iron(III) specific ligands with strong affinity for iron(III) (i.e., at least equal to that of pyrophosphate with a log β = 22.2), including nucleic acids [68] and nucleotide triphosphates, should be considered, although their ability to reach the inner cavity of ferritin is unknown. Smaller ions having lower affinity to iron(III) cations, such as phosphate, lactate, glutamate, or citrate may represent a better option that could assist with the dissolution of the inorganic iron core, similarly to that observed with acetohydroxamate [66]. In this latter case, iron(III) cations, complexed with these small chelates, are released from the interior cavity of ferritin, which upon exiting the ferritin shell encounter the stronger chelate agents (i.e., nucleotide triphosphate), followed by complexation and reduction to iron(II). Such process could constitute a futile cycle for the release of small amounts of iron cations, to help maintain the labile iron pool, under conditions that do not require large spikes in iron protein synthesis.

## 7. Conclusions and Perspective

Under anaerobic conditions, the reductive mobilization of iron from ferritin, in cell free systems, proceeds via electron transfer reactions across the protein shell. This process is likely to be physiologically relevant in anaerobic bacteria, where ferredoxin serves as the electron source, but its relevance under aerobic conditions is unknown. Reduced flavins are one-electron reducing agents that react much more rapidly with molecular oxygen than with the ferritin iron core, and are unlikely relevant iron reducing agents in oxygenated cells. Although some phenazine derivatives have been described as more efficient electron transfer mediators that facilitate the mobilization of iron from ferritin, even in the presence of dissolved oxygen [68], the exact mechanism of this electron mediation process is unclear. Additionally, the efficiency of these mediators in the presence of different concentration of molecular oxygen, and the relevance of this mechanism in vivo, remains to be determined. Nonetheless, this potential chemoselectivity for ferritin iron core reduction vs. oxygen reduction, is an attractive possibility that could support an auxiliary iron mobilization mechanism, in addition to ferritin proteolytic degradation. Other endogenous compounds including oxidoreductases are also worth exploring as potential ferritin-reducing agents.
